# Intragenomic conflict in populations infected by Parthenogenesis Inducing *Wolbachia *ends with irreversible loss of sexual reproduction

**DOI:** 10.1186/1471-2148-10-229

**Published:** 2010-07-28

**Authors:** Richard Stouthamer, James E Russell, Fabrice Vavre, Leonard Nunney

**Affiliations:** 1 Department of Entomology, University of California, Riverside, CA 92521, USA; 2 Université de Lyon; Université Lyon 1; CNRS; UMR 5558, Laboratoire de Biométrie et Biologie Evolutive, 43 Boulevard du 11 Novembre 1918, Villeurbanne F-69622, France; 3 Department of Biology, University of California, Riverside, CA 92521, USA

## Abstract

**Background:**

The maternally inherited, bacterial symbiont, parthenogenesis inducing (PI) *Wolbachia*, causes females in some haplodiploid insects to produce daughters from both fertilized and unfertilized eggs. The symbionts, with their maternal inheritance, benefit from inducing the production of exclusively daughters, however the optimal sex ratio for the nuclear genome is more male-biased. Here we examine through models how an infection with PI-*Wolbachia *in a previously uninfected population leads to a genomic conflict between PI-*Wolbachia *and the nuclear genome. In most natural populations infected with PI-*Wolbachia *the infection has gone to fixation and sexual reproduction is impossible, specifically because the females have lost their ability to fertilize eggs, even when mated with functional males.

**Results:**

The PI *Wolbachia *infection by itself does not interfere with the fertilization process in infected eggs, fertilized infected eggs develop into biparental infected females. Because of the increasingly female-biased sex ratio in the population during a spreading PI-*Wolbachia *infection, sex allocation alleles in the host that cause the production of more sons are rapidly selected. In haplodiploid species a reduced fertilization rate leads to the production of more sons. Selection for the reduced fertilization rate leads to a spread of these alleles through both the infected and uninfected population, eventually resulting in the population becoming fixed for both the PI-*Wolbachia *infection and the reduced fertilization rate. Fertilization rate alleles that completely interfere with fertilization ("virginity alleles") will be selected over alleles that still allow for some fertilization. This drives the final resolution of the conflict: the irreversible loss of sexual reproduction and the complete dependence of the host on its symbiont.

**Conclusions:**

This study shows that dependence among organisms can evolve rapidly due to the resolution of the conflicts between cytoplasmic and nuclear genes, and without requiring a mutualism between the partners.

## Background

Intragenomic conflicts are a fundamental driving force in evolution [1-3]. Sex and later on anisogamy are thought to have evolved as a consequence of conflicts between selfish genetic elements and their host genome [4]. In turn, sex and anisogamy have promoted other conflicts between cytoplasmic and nuclear genes based on their different modes of inheritance [1]. Cytoplasmic genes are only transmitted through females, rendering males a dead end for them. Therefore, selection at the cytoplasmic level favors any manipulation of the sex ratio increasing female production. In response, nuclear genes are selected to re-establish a balanced sex ratio by suppressing or counteracting the action of cytoplasmic elements. These nucleo-cytoplasmic conflicts can have dramatic consequences on sex-allocation and sex-determination systems [1]. One of the most striking examples of such conflicts is cytoplasmic male sterility (CMS) in hermaphroditic plants where mitochondria promote the production of females by sterilizing male gametes, but are counteracted by nuclear suppressor alleles. It has been proposed that CMS has played a major role in the evolution of dioecy in some plant taxa [5].

Other cytoplasmically inherited genetic elements include endosymbiotic bacteria that are common in many arthropods. In many cases such symbionts cause female-biased offspring sex ratio by several means: either transforming genetic males into females (feminization [6]), killing male offspring [7] or by inducing parthenogenesis [8]. For example; in different strains of *Wolbachia *all of these phenotypes have evolved [9,10], different strains of *Cardinium *have been shown to induce feminization, parthenogenesis and cytoplasmic incompatibility [11-13], while in *Rickettsia *both male killing strains [14] and parthenogenesis inducing strains are known [15]. In the case of feminization, suppressor alleles have evolved and the intragenomic conflict has profoundly affected the sex-determination system in the woodlouse *Armadillidium vulgare *[16]. Male-killers have also been shown to affect sexual selection by reversing the roles of males and females in courtship behaviors [17]. Despite extreme sex ratio biases caused by symbionts, nuclear suppressor alleles do not always evolve rapidly. For instance, the male-killing *Wolbachia *in the butterfly *Hypolimnas bolina *on Independent Samoa has reached extremely high frequencies, only 1 in 100 individuals is male, but nuclear suppressor alleles have not evolved in over 400 generations [18]. However, resistance to male-killing in this system has been shown [19] and a rapid invasion of populations infected with the male killer by these suppressor genes was recently observed [20].

Here we investigate the consequences of nucleo-cytoplasmic conflict when parthenogenesis induction by *Wolbachia *occurs. PI-*Wolbachia *are found in many species of parasitoid wasps and allow infected females to produce daughters from unfertilized eggs [21]. In Hymenoptera the normal, sexual mode of reproduction is such that unfertilized (haploid) eggs become males while fertilized (diploid) eggs develop into females. The PI-*Wolbachia *does not appear to influence meiosis. Instead infected unfertilized eggs become diploid by a *Wolbachia*-induced modification of the first [22,23] or the second mitotic division [24,25]. In all cases studied the outcome is: two identical sets of chromosomes that fail to separate, resulting in egg nuclei that are diploid. These eggs develop into completely homozygous infected females. In *Trichogramma *species the anaphase of the first mitotic division aborts and the resulting diploid individual develops into a female. The ability to develop from an unfertilized egg does not preclude the possibility of fertilization. In fertilized infected eggs, *Wolbachia *does not interfere with normal fertilization. In *Trichogramma *populations, where both infected and uninfected individuals co-exist, infected females mate and produce two types of infected daughters, heterozygous daughters that have a father, and completely homozygous daughters that are parthenogenetically produced [22].

Genetic conflicts have been demonstrated in *Trichogramma kaykai *populations from the Mojave Desert (California, USA). In these populations, the PI-*Wolbachia *is found in all studied field populations at a relatively low frequency of about 10% of females. The infection does not reach higher frequencies because it is countered by the presence of a PSR (paternal sex ratio) chromosome [26]. The PSR chromosome is a B-chromosome that is exclusively transmitted through males. It causes eggs fertilized with sperm from a PSR male to develop into males, instead of females [27,28]. The PSR chromosome accomplishes this by the destruction of the paternal set of chromosomes (excluding itself) in the fertilized egg, therefore the fertilized egg does not develop into a diploid female but becomes a haploid male, again a carrier of the PSR chromosome. The presence of the PSR chromosome in the *T. kaykai *population keeps the *Wolbachia *infection from reaching fixation [26].

In some wasp species the PI-*Wolbachia *infection has gone to fixation in all studied populations [21]. In other species both completely infected ('fixed') and uninfected populations exist geographically isolated from each other [29-32]. 'Mixed' populations, in which infected and uninfected individuals coexists, are only known from several *Trichogramma *species [21]. In practically all 'fixed' populations sexual reproduction appears no longer possible. Males can be derived from such populations by antibiotic treatment and paired with antibiotic treated females, and still no sexual reproduction takes place. In those species where both sexual and infected populations occur in geographically distinct areas, it is possible to investigate which of the two sexes is responsible for the lack of fertilization. In *Apoanagyrus lopezi *[29], *Telenomus nawaii *[30,31], *Leptopilina clavipes *[32], *L. japonica *[33] and *Trichogramma pretiosum *(Peru) [34] males derived from 'fixed' populations by antibiotic treatment are able to inseminate females from sexual populations, and these females use the sperm to successfully fertilize their eggs. However, females from infected populations exposed to males from the sexual population do not fertilize their eggs. Therefore in the 'fixed' infected populations, sexual functionality has been lost in females, but not in males. Similarly, in *Aphytis lignanensis*, *A. diaspidis *[35] and *Eretmocerus mundus *[36] males derived from 'fixed' infected populations produce sperm and mate with infected females, but the females do not then use the sperm to fertilize their eggs. Furthermore, in *Telenomus nawaii*, repeated introgression of nuclear genes from a 'fixed' infected population (using males were derived by antibiotic treatment) into females from an uninfected population, resulted after two generations in the inability of some of the introgressed females to produce fertilized eggs ([31]). This shows that the non-fertilization trait is inherited as one or more nuclear genes.

The loss of female versus male sexual function in 'fixed' infected populations can be explained by selection against female sexual function. Several hypotheses have been proposed to explain this asymmetry in the loss of sexual function. Stouthamer et al [37] posed the "neutral mutation accumulation" hypothesis: once a PI-*Wolbachia *infection had reached fixation there would be no more selection to maintain alleles involved in sexual reproduction and over time both male and female sexual function would erode. Huigens and Stouthamer [21] subsequently suggested that female sexual function would erode faster if more loci were involved in coding for the female behavior than for the male behavior. Alternatively, Pijls et al [29] hypothesized that once a population had reached fixation for the PI-*Wolbachia *infection and sexual reproduction has ceased, those mutations that would disable costly female traits involved with sexual reproduction would be selected. Examples of costly female traits could be pheromone production, maintenance of spermathecal glands etc. This "costly female trait" hypothesis would explain the rapid decline in sexual behavior of the females relative to that of the males. More recently, Huigens and Stouthamer [21] and Jeong and Stouthamer [31] hypothesized that the female-biased sex ratio in populations with a spreading PI-*Wolbachia *infection selects for alleles that increase the production of males, which in haplo-diploids is accomplished by a reduced egg fertilization rate. They used the term "functional virginity mutations" to describe these mutations because the phenotypic result is females that no longer fertilize their eggs. "Functional virginity mutations" could disable any trait required for successful sexual reproduction in females. These same traits could be the target of "costly female trait" mutations. However, in contrast to the "functional virginity" hypothesis, the "costly female trait" hypothesis requires that disabling the trait also results in a positive physiological fitness effect.

Using models, we explore these hypotheses and show that the spread of a PI-*Wolbachia *infection, results in selection favoring mutations in the nuclear genes reducing the female fertilization rate. Our models show that the genetic conflict between PI-*Wolbachia *and the nuclear genome strongly influences offspring sex-allocation and that the final resolution of the conflict is the irreversible loss of sexual reproduction ending in the complete reproductive dependence of the host on its symbiotic counterpart. This is consistent with the irreversible loss of sexual reproduction found in many PI-*Wolbachia *infected species [21].

## Results

### Models

We model the spread of a PI-*Wolbachia *infection in an initially uninfected population. We present these models with increasing complexity. First, we model the case where the *Wolbachia *transmission from mother to offspring is perfect, but where the cost of being infected varies. Next, we introduce imperfect transmission of the PI-*Wolbachia *(in these cases not all the offspring of the infected mothers are infected). We provide analytic solutions for these cases. Finally, we allow the fertilization rate of the females in the population to vary. For this last model we used iteration of numerical examples to provide some exact calculations, and support these results with an approximate general analytic solution.

#### 1. Perfect transmission of the *Wolbachia*

We assume that a parthenogenesis inducing *Wolbachia *enters into a population, has a perfect transmission from mother to offspring, and imposes no cost on infected females so the offspring production of infected females equals that of uninfected females which is the case in some species [38-41]. Assuming the egg fertilization rate of females is *x*, then an uninfected female will produce a fraction of x daughters and 1-x sons, while all the offspring of infected females are daughters (Table 1). The relationship between the fraction of females infected in generation t+1 (I_t+1_) as a function of the infection among females in generation t (I_t_) is:(1)

This can be generalized to(2)

**Table 1:** Proportions of different types of offspring produced by *Wolbachia *infected or uninfected females

	Infected female (Fecundity = ω)		Uninfected mother (Fecundity = 1)	
	
Offspring type	Fertilized eggs (*x*)	Unfertilized eggs (1-*x*)	Fertilized eggs (*x*)	Unfertilized eggs (1-*x*)
Infected daughter	*ωxα*	*ω*(1-*x*)*α*	0	0
Uninfected daughter	ω*x*(1- *α*)	0	*x*	0
Uninfected son	0	*ω*(1-*x*)(1- *α*)	0	1-*x*

The relative offspring production of the infected females compared to uninfected females is *ω*.The transmission efficiency of the *Wolbachia *from infected mother to offspring is α and the egg fertilization rate is *x*. We assume that all females both infected and uninfected have been inseminated.

Under such circumstances the infection will rapidly go to fixation in the population, the speed of the spread being dependent on the fertilization rate. The higher the fertilization rate the longer it will take before the infection reaches fixation.

Infection with the PI-*Wolbachia *may have some physiological cost to the host, resulting in reduced fitness of infected females. In several species, infection with PI-*Wolbachia *reduced the offspring production of infected females relative to uninfected females [36,42-44].

The relative offspring production of the infected females compared to uninfected females equals ω. Under these conditions the infection frequency in generation n is given by:(3)

The dynamics of this system are determined not by the total offspring production but by the number of daughters that the females produce, and only two outcomes are realistic: 1) if an infected female produces fewer daughters than an uninfected female, the infection will not spread (if *ω *<*x*), and 2) if an infected female produces more daughters than an uninfected female the infection goes to fixation (if ω > x).

The number of generations (*n*) it takes to go from an infection frequency among females of I_o _to I_n _is given by:(4)

The time before the infection reaches fixation depends on how much larger ω is than x. It is clear that the number of generations it takes to reach a particular infection frequency increases with a higher fertilization frequency of the eggs, and with a lower relative fitness of the infected females.

#### 2. Imperfect transmission of the PI-*Wolbachia*

The transmission of the *Wolbachia *infection is not perfect in many parasitoid Hymenoptera [21,41,43]. In general, the transmission of *Wolbachia *declines with the age of the mother. The older the infected mother, the more eggs she produces that apparently receive an insufficient dose of bacteria and consequently develop into male offspring. In some species infected females even produce male offspring on the first day of their oviposition [41,45]. Inefficient transmission of the infection can be modeled by defining the fraction of infected virgin female offspring that receive a high enough *PI-Wolbachia *titre to express the parthenogenesis phenotype (i.e. develop into a female) as the transmission efficiency *α *(see Table 1). A mated infected female is expected to produce a fraction of α infected eggs and 1-*α *uninfected eggs. Of the infected eggs a fraction *x *is fertilized and 1-*x *remains unfertilized, but all infected eggs develop into infected females. Of the uninfected eggs a fraction x is fertilized, and develop into uninfected females, and 1-*x *remains unfertilized and develop into males. If the infection also causes a reduced offspring production and all females are assumed to have mated, the infection frequency among females is given by the following recursive relationship:(5)

**Figure 1:**
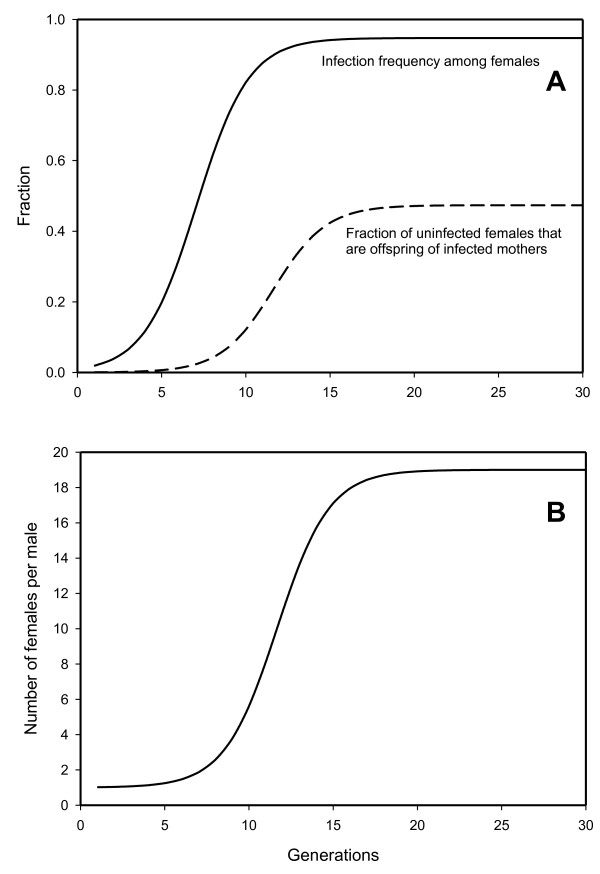
**Spread of PI*-Wolbachia *infection in a population and its effect on the population sex ratio**. A. Solid curve: Increase of PI-*Wolbachia *infection in a population over time where initially 1% of the females are infected, the transmission efficiency of the *Wolbachia *(*α*) is set at 95%; there is no cost of being infected (*ω *= 1). All females are mated. Dashed curve: The fraction of uninfected females that are the offspring of infected mothers. B. The ratio of females to males in the population over the generations. Values were derived using equation 5 from the text.

The PI-*Wolbachia *can only spread from rarity if infected females produce more infected daughters than uninfected females produce uninfected daughters (*αω *>*x*) in which case the infection does not go to fixation but reaches an equilibrium (solid curve Figure 1A). At equilibrium the relationship is given by [45]:
(6)

The equilibrium is maintained because mated infected females also produce some uninfected daughters (Table 1), and this "sponsoring" of the uninfected part of the population by the infected females increases with the infection frequency in the population (dashed curve Figure 1A).

Once the *Wolbachia *infection starts to spread in a population the sex ratio becomes more and more female-biased (Figure 1B). Equation (6) can be used to determine the relative number of females per male (*φ*) for a population at equilibrium:(7)

**Figure 2:**
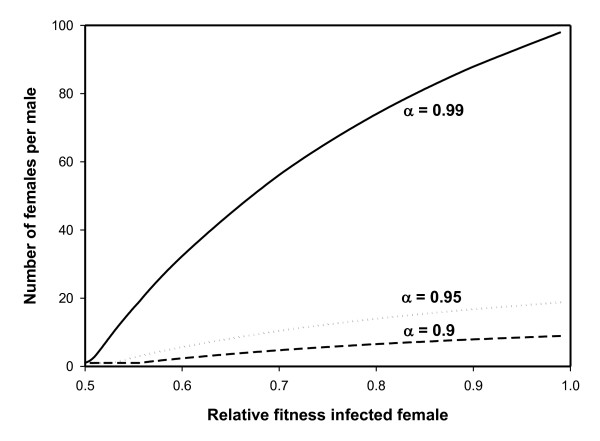
**Effect of PI-*Wolbachia *transmission efficiency and cost of infection on population sex ratio**. The number of females per male in populations where the PI-*Wolbachia *infection is at equilibrium as a function of the relative fitness (offspring production) of the infected females (*ω*) and the *Wolbachia *transmission efficiency (*α*), when the egg fertilization rate is 0.5. Calculations were done using equation 7 from the text.

The degree of female bias increases with the *Wolbachia *transmission efficiency (*α*), and to a lesser extent decrease with the relative cost of being infected (1-*ω*) (Figure 2).

The model integrating the infection cost and, especially, the imperfect transmission is more representative of natural situations. An equilibrium infection frequency can be maintained under these circumstances (equation 6); allowing the long-lasting co-existence of infected and uninfected females and the evolution of conflict between the nuclear and cytoplasmic genes.

#### 3. Evolution of fertilization rate of females

When the population sex ratio becomes more female-biased because of a spreading PI-*Wolbachia *infection, males will have increased fitness due to their high mating rates. Thus alleles that reduce the egg fertilization rate will be selected, since all unfertilized eggs of uninfected females and a fraction (1-*α*) of eggs of infected females become males (see Table 1). Genetic variation for offspring sex ratio (i.e. fertilization rate) has been found in several uninfected parasitoid wasp species [47-49].

We simulated the spread of a PI-*Wolbachia *infection in a population where two fertilization rate variants were present: 1) the wild type fertilization rate (*x*, females expressing this allele fertilize 50% of their eggs) and 2) a recessive mutant fertilization rate allele (*n*, females expressing this allele fertilize a lower percentage of their eggs) using the recurrence relationships defined in additional file 1. The simulations were initiated with a wild type population into which we introduced a PI-*Wolbachia *infection at a 1% frequency among the females (frequency of infected (*I*) wildtype (++) females *I*_++ _= 0.01, frequency of uninfected (*U*) wildtype (++) females *U*_++ _= 0.99, with all other female genotypes at zero; see additional file 1) and a recessive fertilization mutant *n *at a frequency of 1% in the males (*M*_n _= 0.01, *M*_+ _= 0.99).

To confirm the generality of the simulations, we derived an analytic solution for the initial spread or decline of a rare recessive fertilization mutation (additional file 2).

**Figure 3:**
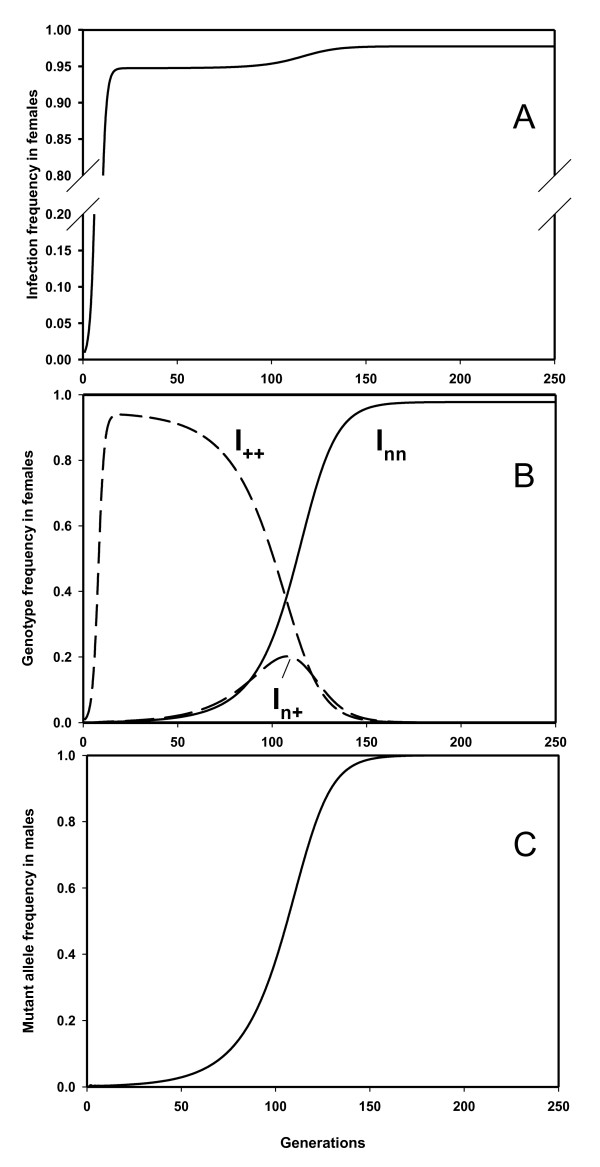
**Spread and subsequent fixation of a low fertilization rate allele in a wild type population**. At generation 1, a PI-*Wolbachia *infection enters the population in 1% of the females, while at the same time the mutant fertilization rate allele (with a fertilization rate of n = 0.3) is entered in 1% of the males. The PI-*Wolbachia *does not have any effect on the offspring production of the infected females (*ω *= 1) and has a transmission efficiency (*α*) of 95%. Genotypes: ++ = homozygote wildtype, nn = homozygote mutant and n+ = heterozygote. Male genotypes either + = wildtype or n = mutant. A. Frequency of *Wolbachia *infection in females; B. Frequency of genotypes among infected females (*I*), note only infected genotype frequencies are displayed.; C. Frequency of the mutant genotype among males, all plotted as a function of the number of generations.

In all simulations the *Wolbachia *infection rapidly spreads through the population to the level determined by the transmission efficiency and the cost of being infected (equation 6). Once the infection has reached high frequencies (Figure 3A) the fertilization rate mutant increased rapidly in frequency and became fixed in both infected and uninfected parts of the population (Figure 3B, C). The increasing frequency of the low fertilization mutant leads to a proportional increase in infection frequency in the population because fewer uninfected daughters are produced because: 1) uninfected females homozygous for the low fertilization mutant produce fewer daughters and 2) infected females homozygous for the low fertilization mutant produce fewer uninfected daughters (reduced sponsoring effect; see Table 1 ). We also demonstrated that spread of the genotype conferring the lowest fertilization rate also occurs within a two-locus model with cumulative effects (results not shown). Each fertilization mutant or combination of mutants, producing the lowest fertilization rate spreads through the population. Given sufficient variation for egg fertilization this ultimately leads to the complete loss of egg fertilization, caused by either the cumulative effect of many mutations at loci each with a small effect on the fertilization rate, or by a single major mutation with a large effect. The end result is a population where all females are infected and no longer fertilize their eggs. During this spread the mating behavior of the males should not be affected since male mating success drives the evolution. Consequently, males are functional and capable of producing offspring with non-mutant females during this period.

The generality of this result is shown by the analytic condition for the initial spread of any recessive allele that reduces the wild type fertilization rate by a fraction *v*, so that the mutant fertilization rate *n *= *x*(1-*v*) (see eqn A2, additional file 2):

where *r *is the prevailing proportion of males in the population. Note that the only necessary condition for the spread is a female biased sex ratio (*r *< 0.5).

**Figure 4:**
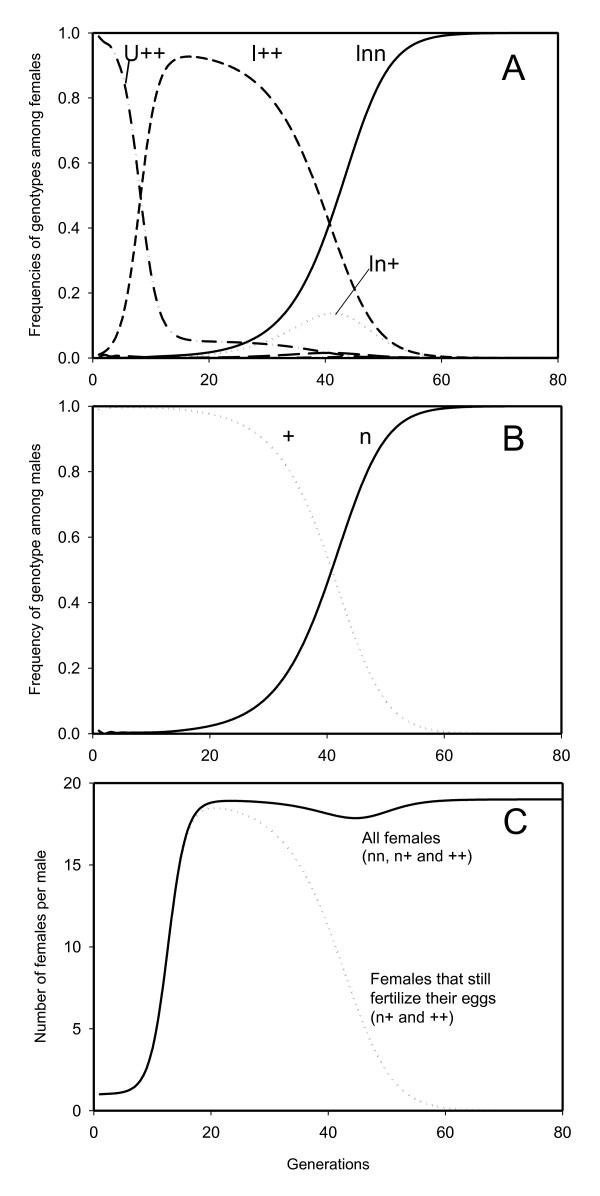
**Spread and subsequent fixation of a virginity mutant in a wild type population**. Spread of the recessive mutant allele for fertilization rate of *m *= 0 ("virginity mutation") in a population with a wild type fertilization rate of *x *= 0.5. In generation 1 a PI-*Wolbachia *infection is entered in the population in 1% of the females, while at the same time the mutant fertilization rate allele is entered in 1% of the males. The PI-*Wolbachia *does not have any effect on the offspring production of the infected females (*ω *= 1) and has a transmission efficiency (*α*) of 95%. Calculations were done using the model described in additional file 1. A: Frequencies of the female genotypes for both infected (*I*) and uninfected (*U*) females, B: Frequencies of the male genotypes and C: Number of females in the population per male for all the female genotypes (nn, n+ and ++; solid line) and for those genotypes that are still fertilizing their eggs (n+ and ++; dotted line)

The mutation(s) causing the lowest fertilization frequency spreads through the population, assuming there are no other fitness costs associated with these mutations. The most extreme case will be a mutant causing females not to fertilize any of their eggs (*n *= 0). Figure 4 shows an example of the changes in genotype frequencies when there is a single fertilization rate locus with two alleles: wild type allele with a fertilization rate of *x *= 0.5 and the recessive mutant allele with fertilization rate *n *= 0. The mutant allele is entered in a wild type population as 1% of the males, simultaneously the PI-*Wolbachia *infection is entered in the population as 1% of the females (see additional file 1). Figure 4 illustrates two aspects of the spread of these fertilization mutants. The mutant allele initially spreads mainly through the selective advantage of males (i.e when the ratio of phenotypically wildtype females to males > 1[dotted line Figure 4c]). However, as soon as this ratio falls below one, there is no longer a selective advantage for the females to produce sons. Although there still is a large ratio of females to males in the population (solid line Figure 4c), an ever decreasing proportion of the females still fertilizes their eggs (dotted line Figure 4c). Yet, the mutation continues to spread in the population, because almost all males carry the mutant allele (solid line Figure 4b). These males are the offspring of infected females homozygous for the mutation. The few remaining phenotypically wildtype females will most likely mate with mutant males. Thus, these two effects together drive the mutation to fixation.

**Figure 5:**
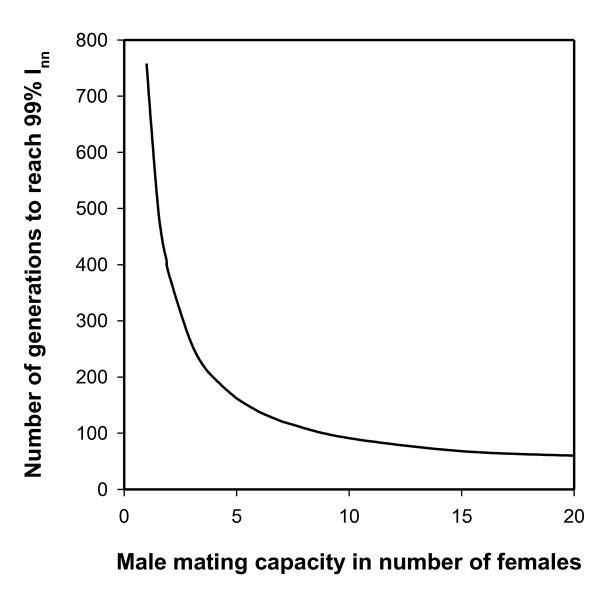
**Speed of fixation for the virginity mutation when male mating capacity varies**. The number of generations required for the recessive mutant allele for fertilization rate of *n *= 0 ("virginity mutation") to spread so that 99% of all females are infected and homozygous for the mutant (*I*_nn_), when the number of females a male is capable of inseminating varies from 1 to 20 females. Calculations were done using the simulation model (additional file 1) with the following conditions: mutant sex ratio allele has a fertilization rate of *n *= 0 in a population with a wild type fertilization rate of *x *= 0.5. In generation 1 a PI-*Wolbachia *infection is entered in the population in 1% of the females, while at the same time the mutant sex ratio is entered in 1% of the males. The PI-*Wolbachia *does not have any effect on the offspring production of the infected females (*ω *= 1) and has a transmission efficiency (*α*) of 95%.

In the previous discussions we assumed that all the females in the population would mate (although not all of them would use the sperm to fertilize their eggs), which may not be realistic given the extremely female biased sex ratio that will be found in the population. It could be argued that if females remain unmated, many more wildtype males are produced and that would slow down or even stop the spread of the fertilization mutants. To simulate the situation where a fraction of the females remains unmated (*p*), we simulated several scenarios first where males had an unlimited mating capacity and all females in the population would be inseminated, next we simulated the spread of fertilization mutation (*n *= 0) when we restricted the males mating capacity (mm) to 15, 10, 5 and 1 female(s) per male respectively. A fraction of females remains unmated (p) if the overall ratio of females to males (R) in that generation is larger than the male mating capacity: p = mm/R (see additional file 3). Figure 5 shows the outcome of these simulations. In all cases even with a very limited male mating ability of 1 female per male both the fertilization mutation and the infection went to fixation, as expected based on our analytic solution, which for this case predicts the spread of a fertilization reducing mutation provided that (see A2, additional file 2):

where *p *is the proportion of females mating, so that, as with full mating, reduced fertilization (*v *> 0) will spread provided the population sex ratio is female biased (*r *< 0.5).

Interestingly in the simulations, the limitation of the male mating capacity resulted in two effects: 1.) the infection frequency went to a much higher level within a few generations, this is caused by a lower production of uninfected females by a.) uninfected mothers- a fraction of them remained unmated and produced only sons, and b.) a fraction of the infected females remained unmated and consequently produced fewer uninfected daughters (reduced sponsoring effect); 2.) in populations with a reduced mating capability it takes longer to reach fixation for the infection and the fertilization mutation because the spread of the mutant allele mainly takes place through the mating of males produced by infected females. And they will only be able to mate with a limited number of females (See legend Figure 5). The mutant allele and the infection finally go to fixation because of a ratcheting effect: all infected females that are homozygous for the fertilization mutation will no longer mate and their female offspring will remain homozygous and infected, yet they will produce male offspring that carries the mutation. This results in most of the males present the population to be carriers of the mutation. Only those females that are not yet homozygous for the mutation will mate and part of their offspring will become homozygous for the mutation. Consequently the class of females that is homozygous for the mutant allele and infected will grow relative to the class of females that is not yet homozygous and infected. Over time this ratcheting mechanism leads to all females being homozygous for the mutation and infected, all the males that are produced in these populations will then also be carriers of the mutation.

Whether the fertilization mutation is dominant or recessive has only little influence on the rate at which the mutation reaches fixation in the population, for most transmission efficiencies the recessive mutant will go to fixation faster than a dominant mutant. Only at very low *Wolbachia *transmission efficiencies does the dominant mutant go to fixation faster (data not shown).

**Figure 6:**
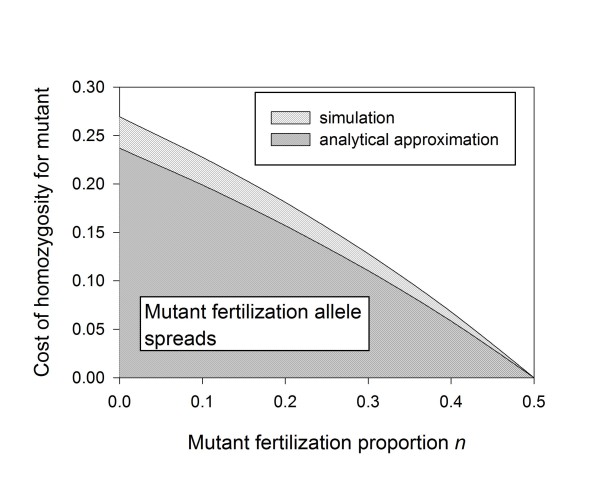
**Mutant fertilization alleles can invade PI-*Wolbachia *infected populations even when they have a negative fitness effect in the homozygous state**. When homozygosity for the recessive mutant allele n with a fertilization rate of *m *also carries a cost (*s*), the mutation can spread from rarity as long as the cost of homozygosity for the mutant and the fertilization frequency of the mutant within in the single hatched (simulation results) or in the double hatched area (simulation and analytical results). The mutant fertilization rate competes with a wildtype fertilization rate of *x *= 0.5. Other values: no cost of being infected (*ω *= 1), the *Wolbachia *transmission efficiency (*α*) equals 0.95.

So far we have assumed that the mutations are neutral with respect to offspring production. In many cases it could be argued that mutations interfering with the fertilization behavior would indeed be neutral or even have a fitness advantage for females. For instance, if the mutation interferes with costly traits involved with sexual reproduction such as pheromone production or storage of sperm etc., the resources that otherwise would have been spent on these traits may become available for other life history traits, resulting in higher offspring production by mutant females. A positive fitness effect of homozygosity for the fertilization mutant allows the mutation to spread more rapidly through the population. However, even a negative fitness effect of homozygosity for the fertilization mutation does not preclude its spread. Using our simulation model (additional file 1) we show in Figure 6 the conditions under which a sex ratio mutant, which when homozygous has a negative fitness effect on its host, is still capable of invading a population with a spreading PI-*Wolbachia *infection. Figure 6 also shows that the simulation results are consistent with the results from our analytic model (eqn A4, additional file 2). The analytic result is consistently slightly more restrictive. This minor difference may be due to the analytic result assumes the spread of the fertilization mutation is only driven by the I_nn _females producing males carrying the mutant allele, while there is at least initially also a small contribution from heterozygous infected and uninfected females.

## Discussion

We show that the female-biased sex ratio caused by a spreading PI-*Wolbachia *infection creates a conflict that gives a large selective advantage to females producing male offspring. The ultimate result of this process is the fixation of mutant alleles reducing the fertilization rate both in the infected and in the uninfected segments of a population. Although our model was based on a single locus, the same principles apply if there are many different loci involved with egg fertilization, thus selection should eventually lead to the fixation of mutant alleles at many loci or to fixation of a mutant allele with a major effect at a single locus, in either case resulting in no egg fertilization at all. Fixation of these mutant alleles occurs even though above a certain frequency there will no longer be an advantage to producing males (since only a small proportion of the females would still be fertilizing their eggs). The spread continues because by that time most of the males in the population will carry the mutation and the some of the daughters of females still willing to mate- i.e. those females not homozygous for the mutation- will become homozygous for the mutation (Figure 4). The ultimate outcome is fixation of both PI-*Wolbachia *infection and the mutations interfering with sexual reproduction (egg fertilization).

If males are produced in these 'fixed' populations, they are in principle still capable of mating and producing sperm, but it is to be expected that females will no longer fertilize their eggs, and as a consequence, sexual reproduction cannot be regained in these populations even if the PI-*Wolbachia *infection is cured. Thus the conflict is resolved by a complete and irreversible loss of sexual reproduction and therefore complete dependence of the host on its symbiont counterpart for reproduction.

While the "costly female trait" hypothesis results in the same outcome as the "functional virginity mutation" hypothesis, their mechanisms differ. The difference between the 'functional virginity mutation" and the "costly female trait" hypotheses is the cost/benefit that is thought to be derived from the mutation. Under the "costly female trait" hypothesis the mutant spreads because females homozygous for the mutation are assumed to be fitter than non mutant females, while no cost assumptions are required under the "functional virginity mutation" hypothesis. Indeed, simulations and an analytical solution (additional file 2) show that even a substantial negative fitness effect does not deter the spread of functional virginity mutations (Figure 6). In addition, because Hymenoptera are known to have mechanisms to adjust their sex ratio, and that some variation exists in natural populations, selection can act readily on fertilization efficiency as soon as *Wolbachia *invades, and without requiring new mutations to arise. Consequently, we expect the "functional virginity" mutations to be the common cause of the loss of sexual function in females from populations fixed for PI-*Wolbachia *infections.

Once *Wolbachia *infection has reached fixation we expect the processes suggested by the other two other hypotheses also to take place. Initially we expect the "costly female traits" mutations to spread through their selective advantage, while other genes involved with sexual behavior in females and all male-specific traits should accumulate mutations at a neutral rate ("neutral accumulation hypothesis"). The longer the population has been fixed for infection the less functional the males are expected to be.

Several changes have been found in the female reproductive organs and behavior in PI-*Wolbachia *fixed species. In most species only the lack of fertilization has been noted and no additional studies have been done to determine if there are morphological or physiological changes in the females or males. One exception to this is the species *Muscidifurax uniraptor *[50] where females lack a spermathecal muscle. In several species where the PI infection has gone to fixation the males have been studied in detail, for instance in *M. uniraptor *males no longer produce sperm. In the species *L. clavipes *the males derived from the infected lines are less fertile than males from an arrhenotokous line [51].

Another effect of this irreversible loss of sexual function is that the fate of the *Wolbachia *and its host are inextricably linked. As a result, selection on the interaction between the *Wolbachia *and its host for increased production of infected offspring by infected females (*αω*) should also become stronger. Such clonal selection can start as soon as infected females have become homozygous for the virginity allele(s). Increased production of infected offspring can be attained by reducing the negative effect of the infection on the offspring production (1-*ω*) and by increasing the transmission efficiency (*α*) of the *Wolbachia*.

After the infection and the virginity mutation(s) have reached fixation, we expect that all females will be identical in those genes associated with the virginity mutations. However, the rest of the genome may differ between different clonal lines. The next selective step in the evolution of PI-*Wolbachia *infected species is a reduction in the number of clones due to selective sweeps favoring reductions in costly female traits and other beneficial mutations, as well as mutations in *Wolbachia *that affect the fitness of infected females. This scenario should result in a large reduction of clonal types in the field, depending on the size of the population and the frequency at which beneficial and costly female trait mutations take place. In addition, we would expect the population to regain some level of clonal diversity in neutral markers between sweeps.

Several fixed PI-*Wolbachia *infected populations have been studied for clonal variation. In *Diplolepis spinosissimae *and *L. clavipes *both fixed infected and uninfected populations were studied for genetic variation, and only a small number of clones were found in infected populations. The number of clones per infected population varied from 1-3 in *D. spinosissimae *as was inferred from three different microsatellite loci [52]. The total genetic diversity in the infected populations was also much lower than in the sexual population [52]. In *Diplolepis rosae *all studied populations were parthenogenetic and most likely infected with *Wolbachia *[53]. Over the whole range (from Sweden to Greece) only 8 different clones were recognized using 9 different allozymes [54]. The situation is less clear in *L. clavipes *where two main clonal types were identified using AFLP markers [32]; but within each clonal type there was considerable genetic variation. Furthermore the genetic variation in the sexual population appeared to be similar to that in the clonal populations.

## Conclusions

Once a PI-*Wolbachia *infection enters in a population several outcomes are possible. If the PI-*Wolbachia *immediately has a perfect transmission the infection will go rapidly to fixation depending on relative offspring production of the infected females. Neither male nor female sexual function is expected to change during this rapid spread of the infection. It is however unlikely that new associations of host and *Wolbachia *will immediately result in a perfect transmission. Many artificial inoculations of *Wolbachia *in novel hosts are unsuccessful, or have a poor transmission [55-59]. If a PI-*Wolbachia *enters a population and it has an imperfect transmission and it allows the *Wolbachia *to spread through the population then several outcomes are possible. Initially such a spread will lead to the coexistence of both infected and uninfected individuals in the population. This prolonged coexistence allows time for several traits to evolve (either by selection on already existing variation or on arising mutations). Here, we have shown that the most likely outcome is the selection for low fertilization mutants in the population, eventually leading to the fixation of the infection in the population and the irreversible loss of sexual reproduction. This appears to be the most common outcome in PI-*Wolbachia *infected Hymenoptera and highlights that dependence among organisms can evolve rapidly due to the resolution of the conflicts emerging between cytoplasmic and nuclear genes, and without requiring mutualism between the partners. This is clearly an alternative to the classical scenarios of evolution of mutualism where dependence is thought to evolve after long co-evolutionary history between partners.

## Methods

### Modeling

The recursive relationships relating the frequencies of the infection and the different fertilization genotypes in present generation to the next are given in Additional file 1. The model assumes non-overlapping generations, and unlimited growth of the population. All females independent of genotype or infection status have an equal chance of mating. These relationships were modeled over the generations using an excel spreadsheet (Additional file 4) that is explained in Additional file 3.

## Authors' contributions

The original idea for this study was conceived by RS and FV. Subsequent models were made by RS and JER. The analytical solution shown in Additional file 2 was derived by LN. The manuscript was written by RS and LN and edited by FV and JER. All authors have read and approved the final manuscript.

## Supplementary Material

Additional file 1 **Recursive equations used in simulations**. Recursive equations used in simulations Click here for file

Additional file 2 **Analytical solution of mutant fertilization allele spread when homozygosity for mutant allele is costly**. Appendix Click here for file

Additional file 3 **Description of the Excel file (see additional file**4**) used in the simulation**. Explanation of the simulation model given in the additional file 4. Click here for file

Additional file 4 **Model used in calculations**. Excel file containing the spreadsheet used in the simulations described in the manuscript. Click here for file
