# Supplementary material for: Intragenomic conflict in populations infected by Parthenogenesis Inducing Wolbachia ends with irreversible loss of sexual reproduction
Source: BMC Evol Biol. 2010 Jul 28;10:229. doi: 10.1186/1471-2148-10-229 (PMC2927591; doi:10.1186/1471-2148-10-229)
Supplement: Additional file 1 — Recursive equations used in simulations. Recursive equations used in simulations [file 1471-2148-10-229-S1.DOC]

Recursive equations used in the model describing the spread of recessive alleles at a locus influencing the fertilization rate of eggs, in a population with both uninfected females and males, and PI-*Wolbachia* infected females. The equations relate the fractions in generation t to generation t+1. The proportion of all females that are infected (*I*) or uninfected (*U*) are subdivided into the three possible genotypes by subscript: ++ indicates a female homozygous for the wild type with a fertilization rate of *x*; *nn* a mutant female with a fertilization rate *n*, and *n+* a heterozygous female with a fertilization rate equal to the wild type *x*. A fraction *p* of the females mate, the remainder (1-*p*) remains unmated. *α* is the transmission efficiency of the *Wolbachia*, and *ω* is the offspring production of an infected female relative to an uninfected female. *s* represents the reduction in offspring production for those females that are homozygous for the mutant fertilization rate. The fraction of males with different genotypes (+ or *n*) is given by *M*_+_ and *M_n_* respectively.

Total females in generation t+1: *W_f_* = *p*(*I_nn(t)_* (1-*s*)+*I_n+(t)_*+*I_++(t)_*)*αω* + *pI_nn(t)_*(1-*s*)(1-*α*)*ωn* + *p*(*I_n+(t)_*+*I_++(t)_*)(1-*α*)*ωx* + (1-*p*)(*I_nn(t)_* (1-*s*)+*I_n+(t)_*+*I_++(t)_*)*αω* + *pU_nn(t)_*(1-*s*)*n*+ *p*(*U_n+(t)_* + *U_++(t)_*)*x*

Total males in generation t+1: *W_m_*= *pI_nn(t)_*(1-*s*)(1-*α*)*ω*(1-*n*) + *p*(*I_n+(t)_*+*I_++(t)_*)(1-*α*)*ω*(1-*x*) + (1-*p*)(*I_nn(t)_*(1-*s*)+*I_n+(t)_*+*I_++(t)_*)(1-*α*)*ω* + *pU_nn(t)_*(1-*s*)(1-*n*) +*p*(*U_n+(t)_* + *U_++(t)_*)(1-*x*)+ (1-*p*)(*U_nn(t)_* (1-*s*)+*U_n+(t)_* + *U_++(t)_*)
