# Supplementary material for: Intragenomic conflict in populations infected by Parthenogenesis Inducing Wolbachia ends with irreversible loss of sexual reproduction
Source: BMC Evol Biol. 2010 Jul 28;10:229. doi: 10.1186/1471-2148-10-229 (PMC2927591; doi:10.1186/1471-2148-10-229)
Supplement: Additional file 2 — Analytical solution of mutant fertilization allele spread when homozygosity for mutant allele is costly. Appendix [file 1471-2148-10-229-S2.DOC]

**Analytical solution for spread of a virginity mutant from rarity:**

To determine the fate of a virginity allele, we can consider the spread of a new recessive mutation that decreases the fertilization rate in a population infected with PI-*Wolbachia*. Initially there are no fitness consequences for the new mutation, since it is recessive in females and has no effect in males. In a random mating population, if the population was not infected by *Wolbachia*, the creation of a homozygous female would be very unlikely; however, the creation of homozygotes of a rare allele is always rapid in the presence of *Wolbachia*.

Consider the possible initial conditions: the mutation may occur in a male (case 1); in a female, creating either an uninfected heterozygote (case 2), or an infected heterozygote (case 3). In case 1, the male’s mutation can only be inherited by a female, and she will be a heterozygote who is either uninfected (creating case 2) or infected (creating case 3) depending upon his mate. In case 2, the heterozygous uninfected female can pass her mutation on to a son (creating case 1) or to an uninfected heterozygous daughter (recreating case 2). In case 3, the important transition occurs: a fraction *α*(1-*x*)/2 of the offspring are homozygous for the novel allele. These homozygotes are created when the female fails to fertilize an egg (with probability 1-*x*) that carries the mutation (with probability of 0.5), and the *Wolbachia* infection is retained (with probability *α*), so the haploid egg becomes diploid and homozygous. There are no fitness differences relating to the new mutant allele generated in any of these three cases.

Once the homozygous mutant has been created then it is the fitness of that genotype that primarily drives the spread or elimination of the mutant. These dynamics are complex because of the frequency dependent effects inherent in any system dependent upon mating frequencies. However, given random mating, we can get a good approximation of the dynamics by considering the fitness of an infected homozygous mutant female, in terms of her production of grand-offspring:

*W_Inn_* = (Fecundity)[daughters + males(matings /male)]


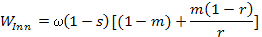


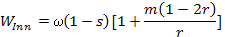


where ω is the offspring production of an infected female relative to an uninfected female, *s* is the cost of the new mutation, *r* is the sex ratio of the whole population, *m* is the proportion of sons produced by the infected homozygous females (genotype *Inn*). The production of sons is defined by:


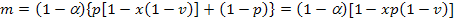


where *p* is the probability of mating, α is the probability of transmission of *Wolbachia*, *x* is the established fertilization rate and *v* is the proportional reduction in the rate of fertilization in the homozygous mutant female. The mutant fertilization rate *n* = *x*(1-*v*)

In general, does fitness of the *Inn* genotype increase with *v*?


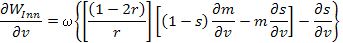


where:


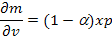


And
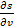
 defines the relationship between the fitness loss and the reduction in fertilization rate . In the absence of any cost to a lowered fertilization rate, *s* = 0, and we have


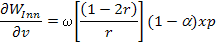


Thus in the absence of a cost, lowering of the fertilization rate (by increasing *v*) is always favored (
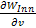
 > 0), regardless of the prevailing fertilization strategy *x*, provided the proportion of males in the population is under 50%. This result also shows that the fitness advantage of reduced fertilization is dependent upon the production of males by infected females, as defined by (1-α) (see table 1).

If there is a loss of fitness (*s*>0) associated with reducing the fertilization rate, then whether or not the optimum strategy is to fully minimize the fertilization rate (*v* = 1) depends upon the unknown nature of
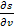
.

Without knowing the general trade-off relationship
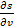
 between fertilization rate and cost (if one exists), we can instead compare the fitness difference between the current strategy and some new (rare) strategy with known fertilization rate *n*= (*x*(1-*v*)) and cost *s.* The new rare mutant spreads provided that:


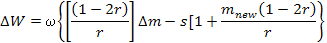


Where
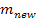
 is the production of sons by females homozygous for the new rare mutant and Δ*m* is the increase this represents over the original level.


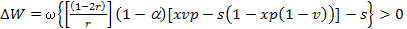
 A1

since
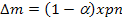
. Given no cost (*s* = 0) this simplifies to:


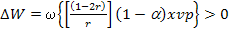
 A2

From (A1) and (A2) can be seen that an important determinant of the outcome is the prevailing sex ratio, defined by eqn (6) for p = 1:


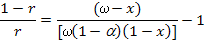


This leads to the condition that, for *p*=1, a new mutant will spreads provided that:


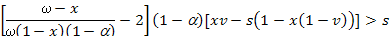


If we assume *ω* = 1, *x* = 0.5 then the mutation will spread from rarity provided:


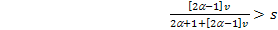
 A3

For α=0.95, we have 0.9*v*/(2.9+0.9*v*) > *s,* so a cost of 0.237 can be tolerated given a fertilization rate of zero (*v* = 1), while for α=0.5, no cost can be tolerated (*s*=0 for *v*=1). In all cases tested by simulation, this condition predicts a slightly lower maximum cost than the full model permits, i.e. it is conservative with respect to the ultimate fate of the fertilization mutant (see fig 6).
