# Supplementary material for: Intragenomic conflict in populations infected by Parthenogenesis Inducing Wolbachia ends with irreversible loss of sexual reproduction
Source: BMC Evol Biol. 2010 Jul 28;10:229. doi: 10.1186/1471-2148-10-229 (PMC2927591; doi:10.1186/1471-2148-10-229)
Supplement: Additional file 3 — Description of the Excel file (see additional file4) used in the simulation. Explanation of the simulation model given in the additional file 4. [file 1471-2148-10-229-S3.DOC]

Description of the excel model used in the calculations.

The spreadsheet is organized such that the first set of columns (B-I) give the present population composition for the different infections types (infected indicated by I, and uninfected by U) the different genotypes are indicated by nn= homozygote mutant sex ratio, n+ = heterozygote and ++= homozygote wildtype; the males (M) are all uninfected and haploid and they are either mutant (n) or wildtype (+). The second set of columns (J-Q) consists of the calculations for the next generation indicated by the prefix New. Columns R and S contain respectively the total number of females and males. Each generation the total number of females is normalized to one, and consequently the different classes of females sum to one after normalization. This is done in the first set of columns (B-G) of the next generation where the number of females in the different classes calculated in the previous generation as the New generation is divided by the total number of females of that generation (column R). The same is done for the males in columns M and N. To determine the fraction of the females that remains unmated we calculate p in column Y. If the total number of females divided by the total number of males (see column W) is higher than the male mating capacity (mm see cell U8), then the fraction of females that remains unmated p is equal to the fraction male mating capacity (mm) divided by the number of females per male (column W). Alternatively if there are fewer females per male than the male mating capacity (mm) then the value of p is set to one and all females mate. Columns U and V give the names and the values for the different variables, with x being the wildtype fertilization rate, xn the mutant fertilization rate, p the value for p used in the calculation for the first new generation, in the subsequent generations the calculated values of p are given in column Y. Alpha is the transmission efficiency of the *Wolbachia* by infected females to their offspring. The offspring production of an infected female relative to an uninfected female is w. Cost is the cost of being homozygous for the mutant sex ratio, this cost is only assessed in homozygous mutant females. In column X the number of females, that are not expressing the mutant fertilization rate, per male are calculated. Column Z gives the fraction of uninfected females that is the offspring of infected mothers. Column AB and AC gives the non normalized numbers of mutant males that is the offspring of resp. infected and uninfected mothers, and column AC gives the ratio of mutant males produced by infected and uninfected mothers. Column AD represents the fraction of all females that is infected.
